# Bistatic ISAR Imaging with a V-FM Waveform Based on a Dual-Channel-Coupled 2D-CS Algorithm

**DOI:** 10.3390/s18093082

**Published:** 2018-09-13

**Authors:** Jiyuan Chen, Xiaoyi Pan, Letao Xu, Wei Wang

**Affiliations:** 1 State Key Laboratory of Complex Electromagnetic Environment Effects on Electronics and Information System, National University of Defense Technology, Changsha 410073, China; mrpanxy@nudt.edu.cn (X.P.); 13807319968@139.com (W.W.); 2 Naval Research Academy, PLA (NVRA), Beijing 100000, China; xuletaowin@126.com

**Keywords:** V style modulation (V-FM) waveforms, bistatic inverse synthetic aperture radar (Bi-ISAR), two-dimension compressed sensing (2D-CS)

## Abstract

Due to the sparsity of the space distribution of point scatterers and radar echo data, the theory of Compressed Sensing (CS) has been successfully applied in Inverse Synthetic Aperture Radar (ISAR) imaging, which can recover an unknown sparse signal from a limited number of measurements by solving a sparsity-constrained optimization problem. In this paper, since the V style modulation(V-FM) signal can mitigate the ambiguity apparent in range and velocity, the dual-channel, two-dimension, compressed-sensing (2D-CS) algorithm is proposed for Bistatic ISAR (Bi-ISAR) imaging, which directly deals with the 2D signal model for image reconstruction based on solving a nonconvex optimization problem. The coupled 2D super-resolution model of the target’s echoes is firstly established; then, the 2D-SL0 algorithm is applied in each channel with different dictionaries, and the final image is obtained by synthesizing the two channels. Experiments are used to test the robustness of the Bi-ISAR imaging framework with the two-dimensional CS method. The results show that the framework is capable accurately reconstructing the Bi-ISAR image within the conditions of low SNR and low measured data.

## 1. Introduction

The inverse synthetic aperture radar (ISAR) has many important detecting and recognizing applications in many civilian and military fields such as air traffic control, harbor and river traffic surveillance, and the remote sensing of satellites under the conditions all-weather and all-day [1,2,3]. Compared to monostatic ISAR, Bistatic ISAR (Bi-ISAR) has many advantages, such as platform flexibility, and immunity to electronic countermeasures and physical attack. The configurations in which the transmitter and the receiver are installed on separate platforms can offer more freedom for acquiring complementary target information and avoiding blind velocities [4,5,6]. In conventional Range-Doppler imaging, by means of intra-pulse frequency modulation for radar waveforms, the range resolution and the detection range can be improved via the pulse compression technique and large time-bandwidth product waveforms [7]. These large time and band product signals, which include chirp signal, frequency-stepped signal, and frequency-stepped chirp signal, are commonly utilized in modern radar systems [8,9,10,11]. However, the drawback of the chirp signal is that the ambiguity apparent in range and velocity, which can be seen from the “ridge” ambiguity function, and frequency-stepped waveform need a relatively long coherent processing interval (CPI) to complete the transmitting and receiving process of each burst, and the wide aspect angle with long CPI may lead to range cell migration (RCM). These disadvantages greatly degrade the ISAR imaging quality. Thus, ISAR imaging based on the V-style modulation (V-FM) waveform has been proposed because of the “thumbtack” ambiguity function, which can mitigate the ambiguity apparent in range and velocity [12].

Compressed Sensing (CS) theory states that sparse or compressible signals are able to be reconstructed exactly from limited measurements by solving a sparsity-constrained optimization problem with a high probability, which has been used in many fields such as medical imaging and radar [13]. CS has been adopted to achieve high resolution and well-focused ISAR images using sparse sampling data [14,15,16]. ISAR imaging with wideband V-FM waveforms has been proposed in [17]. Gu and Pan et al. in [12] adopted dual-channel CS-based dechirping (CS-D) to achieve high resolution range profiles (HRRPs) of V-FM waveforms. In the framework, the HRRPs reconstruct themselves by solving the a $l_{1}$ norm convex optimization problem and using Fourier transform (FT) in azimuth direction to obtain the final 2D images. An improved CS-based high azimuth imaging method is developed to overcome strong noise and clutter in [18]. However, the above methods are the sparsity-based high range or azimuth resolution imaging methods, which are very time consuming because of the high computational complexity and storage system requirements. To avoid the above disadvantages, two-dimension (2D) coupled imaging methods have been proposed [19,20,21,22]. The traditional 2D Fourier Transform method (2D-FFT) needs long data collection, which in reality, is unachievable. In [19], sensing matrices are designed in the cross range and range direction to implement sparse sampling in both directions and 2D-SL0 algorithm is used to achieve high-resolution, fully polarimetric ISAR images. A novel two-dimensional (2D) group primal dual active set with the continuation (2DGPDASC) algorithm is used to recover an ISAR image in [10]. In [22], the sparsity-based imaging algorithms used to achieve range and azimuth resolutions simultaneously are proposed, but the ISAR imaging matrix vectoring to the one-dimensional (1D) model results in extensive memory usage.

In summary, existing research on ISAR imaging with the V-FM signal can achieve high-resolution images based on 1D sparsity-driven algorithms, but there is little research on V-FM signal Bi-ISAR 2D-CS imaging. In this framework, we establish Bi-ISAR echoes as a 2D-coupled, super-resolution model in the range and azimuth domains by analyzing the characteristics of echoes. Then, we adopt a dual-channel, two dimensional, smoothed L0 (2D-SL0) model to achieve Bi-ISAR imaging, which belongs to a nonconvex optimization problem. Using the steepest descent method and gradient projection principle, the nonconvex approach is advantageous in that it usually yields sparser solutions for a given size of samples, as well as short reconstruction time and low calculation load.

The remainder of this paper is organized as follows. In Section 2, the signal model and Bi-ISAR echoes are introduced. The CS theory is presented in Section 3, the 2D-coupled SR models of V-FM echoes in the dual channel are constructed, and the improved dual-channel 2D-SL0 algorithm for Bi-ISAR imaging is proposed. Section 4 presents the experimental results to validate the effectiveness of the proposed method, and, finally, conclusions are drawn and presented in Section 5.

## 2. Signal Model and Imaging

**Figure 1:**
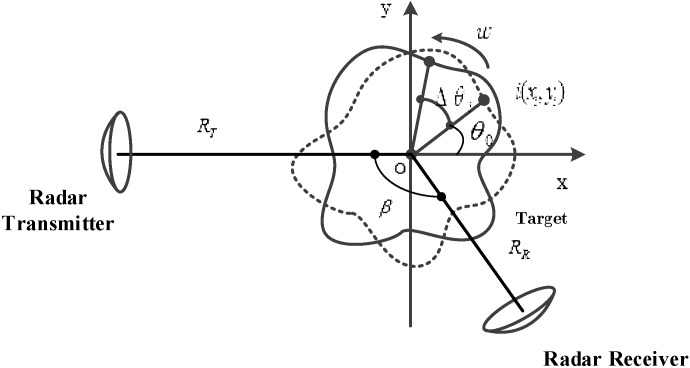
ISAR (Inverse Synthetic Aperture Radar) imaging geometric model.

The relative motion between radar and target in ISAR imaging consists of translation motion and rotation motion. In this paper, we suppose that the target’s translation motion, which is defined as the movement of the target along the range axis of the radar, has been well completed previously [2]. The target has been converted into a turntable model. The geometry of bistatic ISAR imaging is illustrated in Figure 1, the local coordinate system. xoy is on the moving target, and the point o of the moving target is chosen as the origin. The moving target has the circular motion with a constant rotation rate of w rad/s; $r_{i}$ represents the distance from point i to reference point o, and $R_{R}$ and $R_{T}$ are the distances from point o to the receiver and transmitter, respectively. The dash line represents the initial position of the target, the solid line represents the position after a rotation angle $\Delta \theta_{i}$, and β is the bistatic angle, which is kept constant during short CPI; the instantaneous distance sum from the scattering point i to the transmitter and receiver is given as (see Appendix A.1)

(1) $$R\approx R_{T}+R_{R}+2\cos\frac{\beta }{2}\left[y_{i}\cos\Delta \theta_{i}+x_{i}\sin\Delta \theta_{i}\right]$$

Generally, the observation time of Bi-ISAR imaging is very short, and the rotation angle can be approximated by the first-order series

(2) $$R\approx R_{T}+R_{R}+2\cos\frac{\beta }{2}\left[y_{i}+x_{i}wt_{m}\right]$$

**Figure 2:**
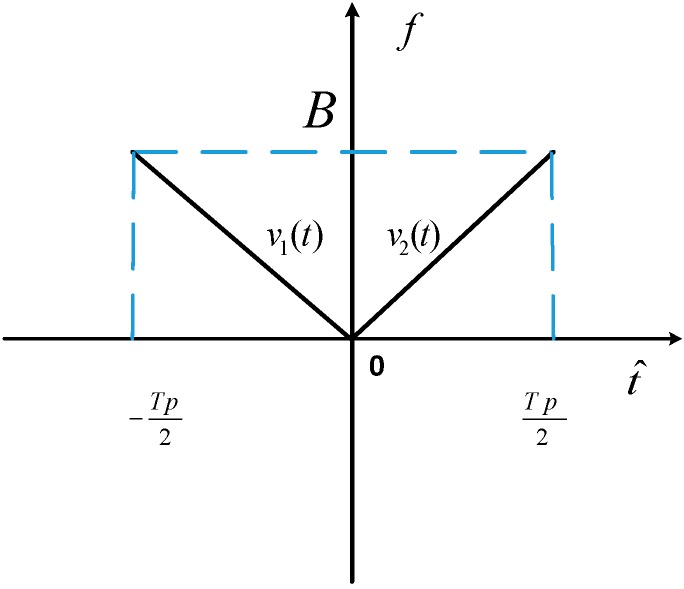
Sketch map of V-FM (V style modulation) waveforms.

Assume that the V-FM waveforms are composed of two chirp signals with opposite slopes as depicted in Figure 2, which follows

(3) $$\upsilon \left(\hat{t}\right)={\mathrm{rect}}_{half}^{-}\left(\frac{\hat{t}}{T_{p}}\right)\upsilon_{1}\left(\hat{t}\right)+{\mathrm{rect}}_{half}^{+}\left(\frac{\hat{t}}{T_{p}}\right)\upsilon_{2}\left(\hat{t}\right)$$

in which

$${\mathrm{rect}}_{half}^{-}=\left\{ \begin{array}{ll} 1 & \hat{t}\in [-\frac{T_{p}}{2},0]\\ 0 & else \end{array}\right.$$

$${\mathrm{rect}}_{half}^{+}=\left\{ \begin{array}{ll} 1 & \hat{t}\in [0,\frac{T_{p}}{2}]\\ 0 & else \end{array}\right.$$

$$\upsilon_{1}(\hat{t})=\left\{ \begin{array}{ll} \exp\left(-j\pi \gamma {\hat{t}}^{2}\right) & \hat{t}\in [-\infty ,0]\\ 0 & else \end{array}\right.$$

$$\upsilon_{2}(\hat{t})=\left\{ \begin{array}{ll} \exp\left(j\pi \gamma {\hat{t}}^{2}\right) & \hat{t}\in [0,\infty ]\\ 0 & else \end{array}\right.$$

and γ is the chirp rate, $T_{p}$ is the pulse duration and bandwidth $B=\gamma T_{P}$, and $\hat{t}$ represents the fast time. Figure 2 shows the time-frequency of V-FM waveforms.

The transmitted V-FM waveform can be expressed as follows:

(4) $$\begin{array}{cc} s\left(\hat{t}\right) & =\upsilon \left(\hat{t}\right)\exp\left(j2\pi f_{0}t\right)\\ & ={\mathrm{rect}}_{half}^{-}\left(\frac{\hat{t}}{T_{p}}\right)\exp\left(j2\pi \left(f_{0}t-\frac{1}{2}\gamma {\hat{t}}^{2}\right)\right)+{\mathrm{rect}}_{half}^{+}\left(\frac{\hat{t}}{T_{p}}\right)\exp\left(j2\pi \left(f_{0}t+\frac{1}{2}\gamma {\hat{t}}^{2}\right)\right) \end{array}$$

in which $f_{0}$ is the frequency of carrier wave, $t_{m}$ is the slow time, t is the full time, and $\hat{t}=t-t_{m}$. If the pulse repetition interval (PRI) of radar is $T_{PRI}$, then $t_{m}=mT_{PRI}$.

**Figure 3:**
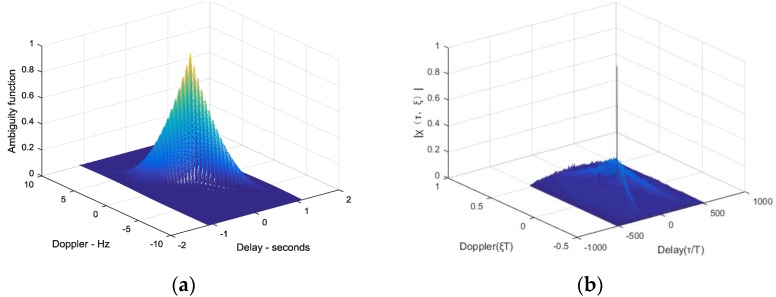
(**a**) Ambiguity function of LFM signal; (**b**) ambiguity function of VFM signal.

To compare the difference between linear frequency modulation (LFM) signal and V-FM signal, the ambiguity function is calculated and simulated, respectively. The definition of the ambiguity function of μ(t) is as follows:

(5) $$\chi \left(\tau ,\xi \right)=\int_{-\infty }^{\infty }\mu (t)\cdot \mu^{*}(t+\tau )e^{j2\tau \xi t}dt$$

in which τ is the delay, which represents range, and ξ is the Doppler frequency, which represents velocity. The results of both signals are shown in Figure 3; the horizontal axis represents range and velocity, and the vertical axis represents the normalized value of |χ(τ,ξ)|, which shows that the “thumbtack” ambiguity function of the V-FM signal mitigates the ambiguity apparent in range and velocity compared with the “ridge” ambiguity function of the LFM signal.

Supposing that the target consists of I strong scatterers, the echo of the scattering center i can be written as

(6) $$\begin{array}{cc} s_{r}\left(\hat{t},t_{m}\right) & =\sum_{i=1}^{K}\sigma_{i}s\left(\hat{t}-\frac{R}{c},t_{m}\right)\\ & =\sum_{i=1}^{K}\sigma_{i}{\mathrm{rect}}_{half}^{-}\left(\frac{\hat{t}-\frac{R}{c}}{T_{p}}\right)\exp\left(j2\pi \left(f_{0}\left(t-\frac{R}{c}\right)-\frac{1}{2}\gamma {\left(\hat{t}-\frac{R}{c}\right)}^{2}\right)\right)\\ & +\sum_{i=1}^{K}\sigma_{i}{\mathrm{rect}}_{half}^{+}\left(\frac{\hat{t}-\frac{R}{c}}{T_{p}}\right)\exp\left(j2\pi \left(f_{0}\left(t-\frac{R}{c}\right)+\frac{1}{2}\gamma {\left(\hat{t}-\frac{R}{c}\right)}^{2}\right)\right) \end{array}$$

in which $\sigma_{i}$ is the scattering coefficient of point i, R represents the distance sum from the *i*-th scattering center to the transmitter and the receiver, and c is the speed of the electromagnetic wave.

The reference signals of dual-channel dechirping can be expressed as

(7) $$s_{ref-1}\left(\hat{t},t_{m}\right)={\mathrm{rect}}_{half}^{-}\left(\frac{\hat{t}-\frac{R_{ref}}{c}}{T_{ref}}\right)\times \exp\left(j2\pi \left(f_{0}\left(t-T_{ref}\right)-\frac{1}{2}\gamma {\left(\hat{t}-T_{ref}\right)}^{2}\right)\right)$$

(8) $$s_{ref-2}\left(\hat{t},t_{m}\right)={\mathrm{rect}}_{half}^{+}\left(\frac{\hat{t}-\frac{R_{ref}}{c}}{T_{ref}}\right)\times \exp\left(j2\pi \left(f_{0}\left(t-T_{ref}\right)+\frac{1}{2}\gamma {\left(\hat{t}-T_{ref}\right)}^{2}\right)\right)$$

in which $R_{ref}$ is the reference range $R_{ref}=R_{R}+R_{T}$, $T_{ref}$ is the reference pulse duration, and $T_{ref}=\frac{R_{ref}}{c}$; then, the echoed signal in dual channels after dechirping can be expressed as follows:

(9) $$s_{if-1}\left(\hat{t},t_{m}\right)=\sum_{i=1}^{K}\sigma_{i}{\mathrm{rect}}_{half}^{-}\left(\frac{\hat{t}-\frac{R}{c}}{T_{p}}\right)\times \exp\left(\frac{j2\pi \gamma }{c}\left(\hat{t}-\frac{R_{ref}}{c}\right)R_{\Delta }\right)\times \exp\left(-\frac{j\pi \gamma }{c^{2}}R_{\Delta }^{2}\right)\times \exp\left(-\frac{j2\pi f_{0}R_{\Delta }}{c}\right)$$

(10) $$s_{if-2}\left(\hat{t},t_{m}\right)=\sum_{i=1}^{K}\sigma_{i}{\mathrm{rect}}_{half}^{+}\left(\frac{\hat{t}-\frac{R}{c}}{T_{p}}\right)\times \exp\left(\frac{j2\pi \gamma }{c}\left(\frac{R_{ref}}{c}-\hat{t}\right)R_{\Delta }\right)\times \exp\left(\frac{j\pi \gamma }{c^{2}}R_{\Delta }^{2}\right)\exp\left(-\frac{j2\pi f_{0}R_{\Delta }}{c}\right)$$

in which $R_{\Delta }=R-R_{ref}$, and we set $R_{ref}=R_{R}+R_{T}$. The first exponentials of $s_{if-1}(\hat{t},t_{m})$ and $s_{if-2}(\hat{t},t_{m})$ are the range items that produce beat frequencies $f_{1i}=\frac{2\gamma R_{\Delta }}{c}$ and $f_{2i}=-\frac{2\gamma R_{\Delta }}{c}$, respectively. The second exponentials of $s_{if-1}(\hat{t},t_{m})$ and $s_{if-2}(\hat{t},t_{m})$ are residual video phases (RVPs), and the third exponentials account for Doppler shift. The RVP can be removed by the translation motion compensation (TMC) algorithm.

Taking the time of reference point as a basis, applying Fourier transform (FT) and multiplying by a constant conversion coefficient 2γ/c, we have

(11) $$s_{if-1}(r,t_{m})=\sum_{i=1}^{K}\frac{T_{p}\sigma_{i}}{2}\sin c\left(\frac{\gamma T_{p}}{c}\left(r-R_{\Delta }\right)\right)\times \exp\left(-j\frac{2\pi f_{0}R_{\Delta }}{c}-\frac{j\pi \gamma {R^{2}}_{\Delta }}{c^{2}}\right)$$

(12) $$s_{if-2}(r,t_{m})=\sum_{i=1}^{K}\frac{T_{p}\sigma_{i}}{2}\sin c\left(\frac{\gamma T_{p}}{c}\left(r+R_{\Delta }\right)\right)\times \exp\left(-j\frac{2\pi f_{0}R_{\Delta }}{c}+\frac{j\pi \gamma {R^{2}}_{\Delta }}{c^{2}}\right)$$

in which r represents the down-range domain. It is noted that two channels have the same amplitude but are symmetrical regarding zero in the down range domain. After envelope alignment and removing the residual video phase (RVP), the final synthesized signal can be obtained as

(13) $$s(r,t_{m})=s_{if-1}(r,t_{m})+fliplr(s_{if-2}(r,t_{m}))=\sum_{i=1}^{K}T_{p}\sigma_{i}\sin c\left(\frac{\gamma T_{p}}{c}\left(r-R_{\Delta }\right)\right)\times \exp\left(-j\frac{2\pi f_{0}R_{\Delta }}{c}\right)$$

in which fliplr(⋅) represents flipping the left part of the signal in a channel to the right part.

Substituting (2) into (13), we have

(14) $$s(r,t_{m})=\sum_{i=1}^{K}\sigma_{i}T_{p}\sin c\left(\frac{\gamma T_{p}}{c}(r-R_{\Delta })\right)\times \exp(j2\pi f_{d}t_{m})\times \exp\left(-j\frac{4\pi }{\lambda }y\cos\frac{\beta }{2}\right)$$

in which $f_{d}=\frac{-2xw\cos\left(\beta /2\right)}{\lambda }$ represents the Doppler frequency and λ is the wavelength. The signal in the range domain, ignoring the constant phase term, becomes

(15) $$s(r,t_{m})=\sum_{i=1}^{K}\sigma_{i}T_{p}\sin c\left(\frac{\gamma T_{p}}{c}(r-R_{\Delta })\right)\times \exp(j2\pi f_{d}t_{m})$$

The cross range compression is obtained by applying FT to (15) in terms of $t_{m}$ as follows:

(16) $$s\left(r,f\right)=T_{p}T_{a}\sum \sigma_{i}\sin c\left(\frac{\gamma T_{p}}{c}(r-R_{\Delta })\right)\times \sin c\left(T_{a}(f-f_{d})\right)$$

in which $f\in [-f_{r}/2,f_{r}/2]$ is the Doppler extent, $f_{r}$ is the pulse repetition frequency (PRF), and $T_{a}$ is the observation time. From (16), it can be seen that the target image is sparse in the Range-Doppler (RD) plane, and there are a few strong scatter points in the whole plane. It can be inferred that RD imaging may fail when measured data is too limited to provide sufficient resolution. Long data-collect time is needed for high resolution in ISAR imaging, but because the targets are noncooperative and usually maneuvering, it is difficult to collect sufficient data and have long observation times, which motivates the study of high-resolution ISAR imaging algorithms based on V-FM waveforms with limited data.

## 3. 2D-CS-Based ISAR Imaging

### 3.1. Review of CS

CS theory provides a new framework for sampling and compressing signals and is able to recover a sparse signal from limited measurements with a high probability. It can overcome the limitation of Nyquist sampling theorem. A signal of interest is expressed as a vector $\alpha \in C^{N}$ which is sparse in some orthonormal basis, and can be represented as α=Ψs, where $\Psi \in C^{L\times L}$ is a linear and orthonormal basis and $s\in C^{L}$ is a sparse vector with less K(K<L) non-zero elements. In other words, the inverse of Ψ represents a sparse transformation that transforms α into a sparse vector s. Suppose that the signal is collected through an inner product of signal with a different sensing matrix, which can be expressed as y=Φα=ΦΨs, in which $\Phi \in C^{Q\times L}(Q<L)$ is known as the measurement matrix, and $y\in C^{Q}$ is a vector of measurement samples. It is worth noting that the number of observations Q cannot be infinitely small, satisfying Q≥O(K⋅lgL). Because Q is much smaller than L, the recovery of the signal s from y is ill-posed. This requires that the dictionary matrix ΦΨ obeys the restricted isometry principle (RIP)

(17) $$\left(1-\delta \right){\left\|s\right\|}_{2}^{2}\leq {\left\|\Phi \Psi s\right\|}_{2}^{2}\leq \left(1+\delta \right){\left\|s\right\|}_{2}^{2}$$

in which δ∈(0,1) is a constant, and ${\left\|\right\|}_{2}$ represents the $l_{2}$ norm. The coefficient α can be exactly recovered from limited measurements by solving the $l_{0}$ norm minimization, which can be described as

(18) $$\left\{ \begin{array}{l} \min\left({\left\|s\right\|}_{0}\right)\;s.t\;{\left\|y-\Phi \Psi s\right\|}_{2}<\varepsilon \\ \alpha =\Psi s \end{array}\right.$$

in which ${\left\|\right\|}_{0}$ represents the $l_{0}$ norm, min(⋅) denotes the minimization, and ε is the noise level. Herein, we can conclude that the CS theory combines three key points to guarantee its performance; the signal to reconstruct is sparse or compressible, the sensing matrix makes ΦΨ so as to satisfy the RIP, and the reconstruction algorithm is efficient and has low computational complexity.

### 3.2. 2D-CS Based ISAR Imaging

According to the practical scattering responses of a target, the final 2D range-azimuth image is always sparse or compressible, since only a few prominent scattering centers occupy the whole imaging plane. We establish the 2D-coupled SR model of the target’s echoes (9) and (10) based on this fact. After the RVP and constant terms are removed, we get dual channels target’s echoes as follows:

(19) $$s_{if-1}\left(\hat{t},t_{m}\right)=\sum \sum_{(x,y)\in I}{\tilde{\sigma }}_{1}\left(x,y\right)e^{-j4\pi \frac{wt_{m}}{\lambda }x}e^{j4\pi \frac{\gamma \hat{t}}{c}y}$$

(20) $$s_{if-2}\left(\hat{t},t_{m}\right)=\sum \sum_{(x,y)\in I}{\tilde{\sigma }}_{2}\left(x,y\right)e^{-j4\pi \frac{wt_{m}}{\lambda }x}e^{-j4\pi \frac{\gamma \hat{t}}{c}y}$$

in which ${\tilde{\sigma }}_{1}(x,y)$ and ${\tilde{\sigma }}_{2}(x,y)$ denotes the complex amplitude of the point $i(x_{i},y_{i})$ in dual channel (see Appendix A.2). In the case of limited bandwidth and small angle, the scattering points do not have Migration Through Resolution Cell (MTRC), so (19) and (20) ignore the influence of $wt_{m}$ on the range image.

The discrete expressions of $s_{if-1}\left(\hat{t},t_{m}\right)$ and $s_{if-2}\left(\hat{t},t_{m}\right)$ are $S_{1}={\left[s_{if-1_{nm}}\right]}_{N\times M}$ and $S_{2}={\left[s_{if-2_{nm}}\right]}_{N\times M}$ (n=0,1,…,N−1; m=0,1,…,M−1), in which N is the number of range samples (range bins) and M is the number of emitted pulses (azimuth samples). Scattering distribution function $\tilde{\sigma }(x,y)$ is discretely represented as a two-dimensional scattering rate distribution matrix $X_{1}={\left[{\tilde{\sigma }}_{1}\left(x_{h},y_{k}\right)\right]}_{N_{r}\times N_{a}}$ and $X_{2}={\left[{\tilde{\sigma }}_{2}\left(x_{h},y_{k}\right)\right]}_{N_{r}\times N_{a}}$ $\left(k=0,1,\ldots ,N_{r}-1;\;h=0,1,\ldots ,N_{a}-1\right)$, in which $N_{r}>N$, $N_{a}>M$. Then, we let $\rho_{x}$ denote the azimuth resolution, $\rho_{y}$ represent range resolution, Δθ represent the imaging accumulation angle, B represent the bandwidth, and Δf represent the frequency sampling interval. Thus, we get the following equation:

(21) $$\begin{array}{ll} x_{h}=h\rho_{x}, & y_{k}=k\rho_{y}\\ \rho_{x}=\lambda /(2\Delta \theta ), & \rho_{y}=c/(2B)\\ B=N_{r}\Delta f, & \gamma \hat{t}=n\Delta f\\ \Delta \theta =wN_{a}T_{p} & \end{array}\}$$

Taking (21) into (19) and (20), we get the discrete form of the ISAR observation signal model

(22) $$S_{1}={\left[s_{if-1_{nm}}\right]}_{N\times M}=\sum \sum_{h,k}{\tilde{\sigma }}_{1_{kh}}e^{-i2\pi \frac{mh}{N_{a}}}e^{j2\pi \frac{nk}{N_{r}}}$$

(23) $$S_{2}={\left[s_{if-2_{nm}}\right]}_{N\times M}=\sum \sum_{h,k}{\tilde{\sigma }}_{2_{kh}}e^{-i2\pi \frac{mh}{N_{a}}}e^{-j2\pi \frac{nk}{N_{r}}}$$

Taking the noise into consideration, the 2D discrete expression ISAR acquisition signals are described as follows:

(24) $$S_{1}=\Phi X_{1}{\Psi^{T}}_{a_{1}}+E$$

(25) $$S_{2}=\Phi X_{2}{\Psi^{T}}_{a_{2}}+E$$

in which $\Phi ={\left[\phi_{0},\phi_{1},\ldots ,\phi_{N-1}\right]}^{T}\in C^{N\times N_{r}}(N<N_{r})$ is the over-complete Fourier dictionaries in the range direction, in which $\phi_{n}=e^{-j2\pi \frac{nk}{N_{r}}}$. $\Psi_{a_{1}}={\left[{\varphi^{-1}}_{0},{\varphi^{-1}}_{1},\ldots ,{\varphi^{-1}}_{M-1}\right]}^{T}$ $\in C^{N_{a}\times M}$, in which $\varphi_{m}=e^{-j2\pi \frac{mh}{N_{a}}}$ and $\Psi_{a_{2}}={\left[\varphi_{0},\varphi_{1},\ldots ,\varphi_{M-1}\right]}^{T}\in C^{N_{a}\times M}$ denote the over-complete Fourier dictionaries of dual channel, respectively, in the cross-range dictionaries. Depicted in the range-Doppler plane, $X_{1}$ and $X_{2}\in C^{N_{r}\times N_{a}}$ represent the interested complex ISAR image, which contains $N_{r}$ range bins and $N_{a}$ Doppler frequency cells. $E\in C^{N\times M}$ denotes the synthetic additive noise matrix. It is worth noting that the popular method to solve the 2D reconstruction problem is to use the Kronecker product and vectorization operation convert 2D-coupled SR model into 1D reconstruction problem. Taking Channel 1 as an example, then (24) is rewritten as $s_{1}=\Theta_{1}x_{1}$, in which $s_{1}=vec\left(S_{1}\right)$, $x_{1}=vec\left(X_{1}\right)$, $\Theta_{1}=\Psi_{a_{1}}⊗\Phi$, and vec(⋅) denotes the vectorization of a matrix, which converts the matrix into a column vector; ⊗ stands for Kronecker product. We note that $\Theta_{1}$ has $NM\times N_{r}N_{a}$ elements, while Φ and $\Psi_{a_{1}}$ is determined by $NN_{r}+N_{a}M$ elements. Thus, the conventional 1D signal model has a large computing memory. This paper focuses on directly solving the 2D-coupled SR model based on a 2D nonconvex optimization problem to avoid this disadvantage.

We solve the ISAR imaging problem as

(26) $$\underset{X_{1}}{\min}{\left\|X_{1}\right\|}_{0}\;s.t.\;{\left\|S_{1}-\Phi X_{1}\Psi_{a_{1}}^{T}\right\|}_{F}^{2}\leq \varepsilon$$

(27) $$\underset{X_{2}}{\min}{\left\|X_{2}\right\|}_{0}\;s.t.\;{\left\|S_{2}-\Phi X_{2}\Psi_{a_{2}}^{T}\right\|}_{F}^{2}\leq \varepsilon$$

(28) $$\hat{X}={\hat{X}}_{1}+{\hat{X}}_{2}={\left[\sigma_{1_{kh}}+\sigma_{2_{kh}}\right]}_{N\times M}$$

in which ${\left\|\right\|}_{0}$ denotes the number of non-zero components in $X_{1}$ and $X_{2}$, ${\left\|\right\|}_{F}$ represents the Frobenius norm of a matrix, and then ε is a small constant.

The optimization problem in (26) and (27) can be solved via 2D-SL0 algorithm described in [23]. SL0 is not based on minimizing the $l_{1}$ norm, but it directly minimizes the $l_{0}$ norm of the solution. It introduces a continuous Gaussian function to approximate the discontinuous $l_{0}$ norm, and then we can get the image X in (28) using a projective steepest ascent approach; the details of this algorithm can be found in [23]. As for V-FM waveform, we give the Two-dimensional SL0 (2D-SL0) Algorithm 1 in channel 1 shown as follows:

**Algorithm 1.** Two-dimensional SL0 (2D-SL0) algorithmInitializationLet ${\hat{X}}_{1_{0}}=\Phi^{†}S_{1}{\left({\Psi^{†}}_{a_{1}}\right)}^{T}$.Choose a suitable decreasing sequence for σ, $\left[\sigma_{1}\ldots \sigma_{J}\right]$.For j=1,…,J:
 (a).Let $\sigma =\sigma_{j}$. (b).Maximize (approximately) the function $F_{\sigma }(X_{1})=\sum_{i,j}\exp(-x_{1_{ij}}^{2}/2\sigma^{2})$ on the feasible set $\left\{X_{1}|S_{1}=\Phi X_{1}\Psi_{a_{1}}^{T}\right\}$ using L iterations of the steepest ascent algorithm (followed by projection onto the feasible set):
-Initialization: $X_{1}={\hat{X}}_{1_{j-1}}$.-For l=1,…,L (Loop L times):(1) Let $\Delta =[\delta_{ij}]$, in which $\delta_{ij}=\exp(-x_{1_{ij}}/2\sigma_{2})$.(2) Let $X_{1}\leftarrow X_{1}-\mu \Delta$ (μ is a small positive constant).(3) Project $X_{1}$ back onto the feasible set:
$X_{1}\leftarrow X_{1}-\Phi^{†}(\Phi X_{1}\Psi^{T}-X_{1}){\left(B^{†}\right)}^{T}$  (c). Set ${\hat{X}}_{1_{j}}=S$.Final answer is ${\hat{X}}_{1}={\hat{X}}_{J}$.in which $\Phi^{†}=\Phi^{T}{\left(\Phi \Phi^{T}\right)}^{-1}$ and ${\Psi^{†}}_{a_{1}}={\Psi^{T}}_{a_{1}}{\left(\Psi_{a_{1}}{\Psi^{T}}_{a_{1}}\right)}^{-1}$ is the pseudoinverse of Φ and $\Psi_{a_{1}}$, respectively. Finally, we can get the ISAR image by (28).

## 4. Experimental Results

**Figure 4:**
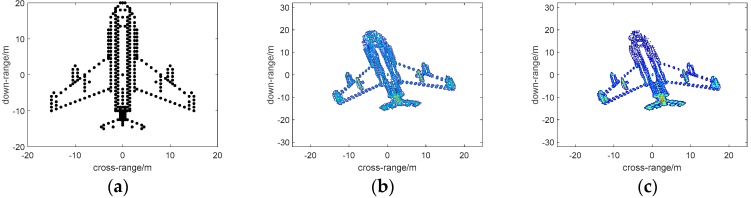
(**a**) Aircraft model, (**b**) RD-based image, and (**c**) 2D-SL0-based image.

In this section, we will implement numerical simulations to verify the performance of dual-channel 2D sparse signal reconstruction in V-FM bistatic ISAR imaging; targets modeled as point scatterers are utilized in the following, as shown in Figure 4a. The V-FM signal ISAR works at the X-band with a carrier frequency of 10 GHz and bandwidth of 300 MHz. The pulse-width is 100 us, the pulse frequency is 1 kHz, and the rotating velocity is 0.05 Hz; we set N=M=256 and $N_{a}=N_{r}=300$. The main simulated parameters are listed in Table 1. The bistatic ISAR imaging results via the traditional RD method and 2D-SL0 are shown in Figure 4b,c; it can be noted that the 2D-SL0 based image shown in Figure 4c has a finer resolution than by another method.

To provide a quantitative evaluation for imaging performance, we take three indexes related to radar target recognition. The first evaluation index is the entropy of the image; the larger the entropy is, the more complex the image information is and the worse the imaging result. The second evaluation index is the normalized root mean square error (RMSE) between the reconstructed image and the target model is computed, which is

(29) $$RMSE=\sqrt{\frac{1}{NM}{\sum_{n=1}^{N}\sum_{m=1}^{M}\left(X(n,m)-\hat{X}(n,m)\right)}^{2}}$$

In addition, the correlation coefficient (CC) is the third index, which can be expressed as follows:

(30) $$CC=\frac{Cov\left(X,\hat{X}\right)}{\sqrt{D(X)}\cdot \sqrt{D(\hat{X})}}$$

in which Cov(⋅) and D(⋅) represent the covariance and variance coefficient between two matrices, respectively.

In the following imaging experiments, these indexes are adopted as the quantitative indexes to evaluate the Bi-ISAR image quality with different reconstruction methods, including 2DFFT, FFT in down-range domain, SL0 in cross-range domain (FFTSL0), and 2DSL0.

### 4.1. Imaging Results Versus Bi-Angle

**Figure 5:**
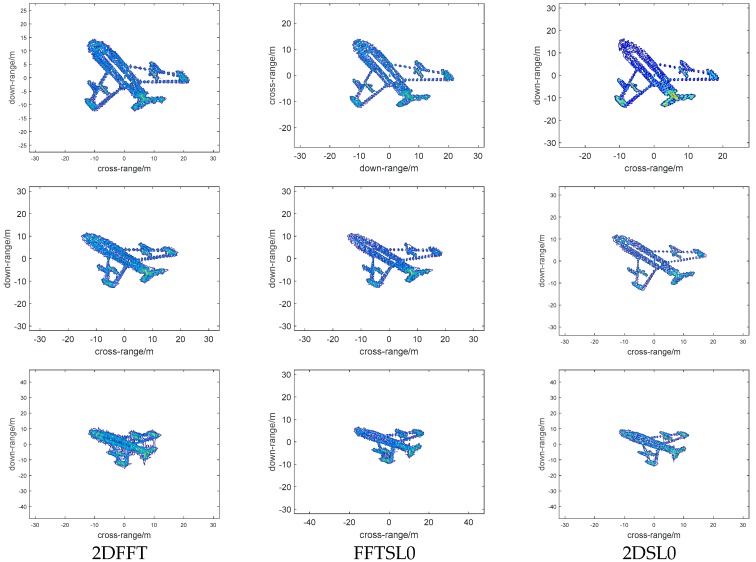
Bi-ISAR (Bistatic ISAR) imaging results with different reconstruction methods versus bi-angle. Column 1 represents 2DFFT images, Column 2 represents FFTSL0 images, and Column 3 represents 2DSL0 images. Row 1 represents bi-angle 60°, Row 2 represents bi-angle 90°, and Row 3 represents bi-angle 120°.

First, we will examine the performance of different reconstruction methods versus bi-angle (60°, 90°, 120°), and the imaging results are shown in Figure 5; the first, second, third columns represent 2DFFT, FFTSL0, 2DSL0 images, respectively, and the first, second, third rows represent images with bi-angle 60°, 90°, and 120°, respectively. We can note that the imaging results become defocused as the bi-angle increases, and 2DSL0 method can provide better focal quality with high resolution than other methods, and, when the bi-angle is 120°, the quality of the image becomes worse. From Figure 5, it implies that the traditional Fourier transform or 2D-CS method cannot eliminate the influence of the bistatic angle and another method is required to overcome this problem.

**Figure 6:**
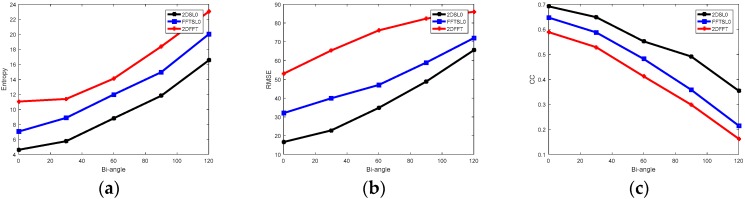
Curves of imaging performance under different bi-angles. (**a**) Entropy versus bi-angle; (**b**) RMSE (root mean square error) versus bi-angle; (**c**) CC (correlation coefficient) versus bi-angle.

To characterize the performance quantitatively, the corresponding curves of entropy, RMSE, and CC are shown in Figure 6. In Figure 6a, it is obvious that the entropy obtained by using 2DSL0 method is lower than that of both 2DFFT and FFTSL0 method. Lower entropy suggests that the quality of imaging result is better. In Figure 6b, the 2DSL0 method performs best among three methods, since it retains the smallest reconstruction errors. From Figure 6c, we find that when the bi-angle is lower than 30°, the reconstructed Bi-ISAR image with the 2DSL0 method is very close to the original image, and CC values increase when bi-angle decreases.

### 4.2. Performance Versus SNRs

**Figure 7:**
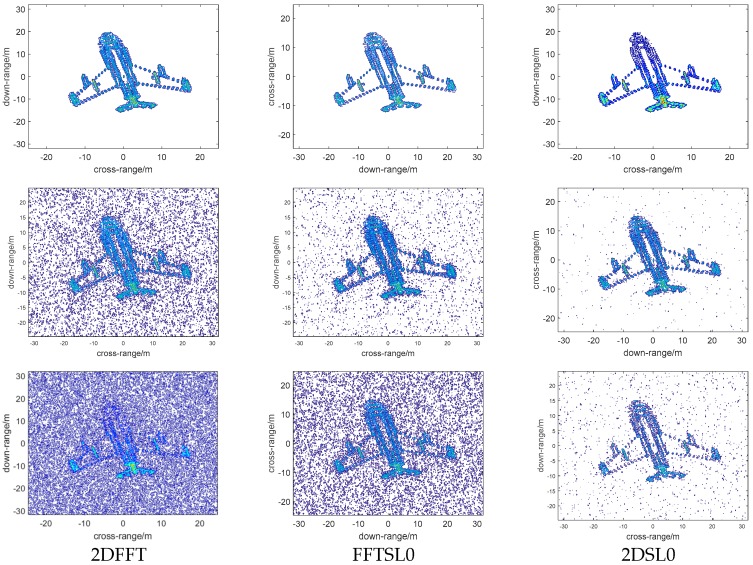
Bi-ISAR imaging results with different reconstruction methods under different SNRs. Column 1 represents 2DFFT images, Column 2 represents FFTSL0 images, and Column 3 represents 2DSL0 images. Row 1 represents SNR is 5 dB, Row 2 represents SNR is 0 dB, and Row 3 represents SNR is −5 dB.

To prove that the robustness of different reconstruction methods degrades with increasing noise, we analyzed the effect of different SNRs on Bi-ISAR imaging; simulations with Gaussian distributed complex noise with three SNRs of 5 dB, 0 dB, and −5 dB added into the simulated VFM signals were performed, and the imaging results are shown in Figure 7. The first, second, and third columns represent 2DFFT, FFTSL0, and 2DSL0 images, respectively, and first, second, and third rows represent images with SNR of 5 dB, 0 dB, and −5 dB, respectively. With the increase in SNR in experiments, the focusing performance of the 2DSL0 method degrades slightly; the 2DSL0 can eliminate much more noise than other methods.

**Figure 8:**
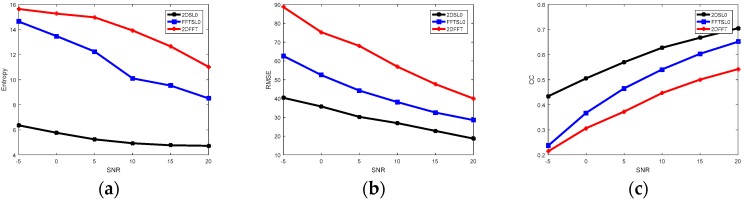
Curves of imaging performance under different SNRs. (**a**) Entropy versus SNRs, (**b**) RMSE versus SNRs, and (**c**) CC versus SNRs.

Entropy, RMSE, and CC are also used to analyze their performance quantitatively, as shown in Figure 8. In Figure 8a, the entropy values of 2DSL0 are much lower than those of both 2DFFT and FFTSL0 method, which means 2DSL0-based imaging results have lower chaos than others methods do, and have the best imaging as a result. In Figure 8b, the 2DSL0 method performs best among three methods, since it retains the smallest reconstruction errors, and the RMSE values decrease under an increase of SNR. From Figure 6c, it notes that when the SNR is higher than 15 dB, the reconstructed Bi-ISAR image with 2DSL0 method is very close to the original image, and the results have a positive correlation with SNR. 

### 4.3. Experiments with Different Echo Data

**Figure 9:**
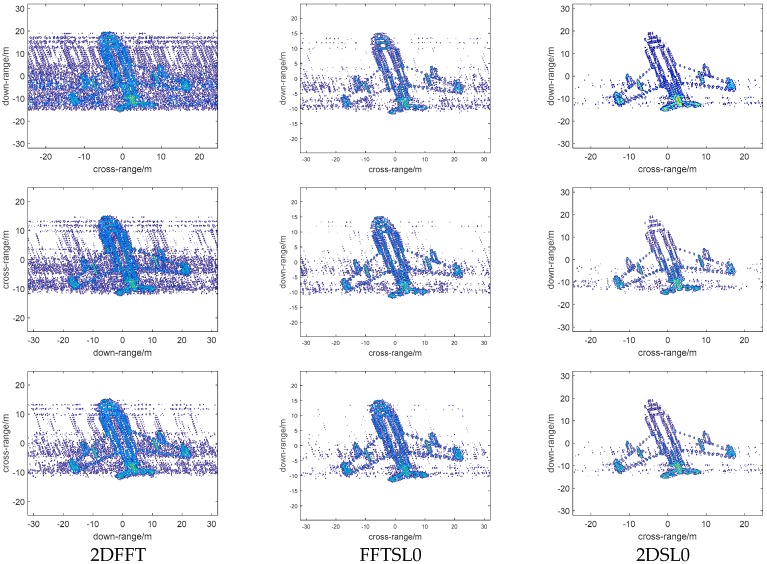
Bi-ISAR imaging results with different reconstruction methods versus measurements. Column 1 represents 2DFFT images, Column 2 represents FFTSL0 images, and Column 3 represents 2DSL0 images. Row 1 represents 50% of echo data, Row 2 represents 65% of echo data, and Row 3 represents 78% of echo data.

In the following, the Bi-ISAR images are generated with different measurements and constant SNR (20 dB), and the measured data used changes from 40% to 80% of echo data. Figure 9 demonstrates the Bi-ISAR imaging results with different reconstruction methods under different measurements (50%, 65%, and 78% of echo data). The first, second, and third columns represent 2DFFT, FFTSL0, 2DSL0 images, respectively, and the first, second, and third rows represent images with measurements (50%, 65%, and 78% of echo data, respectively). It is noticeable that among these Bi-ISAR images, the 2D resolution and focusing energy obtained by the 2DSL0 method is the best, and the 2DFFT method the worst. There is aliasing in cross-range domain under incomplete measurements when using traditional Fourier transform.

**Figure 10:**
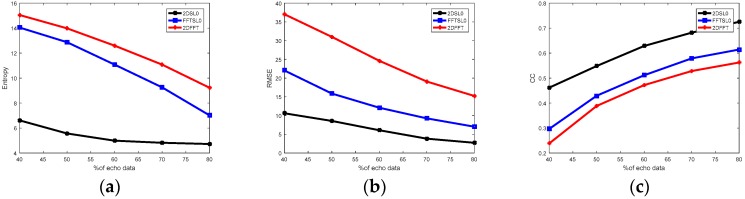
Curves of imaging performance under different measurements. (**a**) Entropy versus measurements, (**b**) RMSE versus measurements, and (**c**) CC versus measurements.

Taking three indexes to analyze the quality of imaging results with different reconstruction versus measurements, as shown in Figure 10. In Figure 10a, the entropy values of 2DSL0 are much lower than those of both 2DFFT and FFTSL0 method, especially when the measurements of echo data are low. In Figure 8b, the 2DSL0 method performs best among three methods, since it retains the smallest reconstruction errors, and more echo data provides more information for imaging. In Figure 10c, the CC values of the 2DSL0 method are larger than those both methods. The larger CC is higher than the similarity between the reconstructed Bi-ISAR image and the referenced image. This implies that the 2DSL0 method can perform more accurate reconstruction than the other two methods.

## 5. Conclusions

In this paper, based on the characteristics of the V-FM signal, we have proposed a dual-channel 2D bistatic ISAR imaging method, which has different dictionaries in each channel and solves a nonconvex optimization problem that can be handled efficiently and reliably. By analyzing the signal and image model of VFM signal bistatic ISAR via traditional Fourier transform and then establishing the 2D SR model of the target’s echo, one can reconstruct the images using the 2D-CS method. Using the simulated data of an aircraft to validate the 2DSL0 based imaging processing, we show that the proposed method maintains better performance than other methods in a low SNR environment and with low measurement ratios of echo data.

## Figures and Tables

**Table 1:** Simulated parameters.

$f_{0}$	10 GHz	$R_{T}$	500 km
B	300 MHz	$R_{R}$	600 km
$T_{p}$	100 μs	β	30°
PRF	1 kHz	w	0.05 Hz

## Appendix A

### Appendix A.1. Derivation of Instantaneous Range in (1)

During short CPI, assume bistatic angle remains constant, the instantaneous distance in Figure 1 can be represented as

(A1) $$R=\sqrt{R_{T}^{2}+r_{i}^{2}-2R_{T}r_{i}\cos\left(\frac{\pi }{2}+\frac{\beta }{2}+\theta_{i}\right)}+\sqrt{R_{R}^{2}+r_{i}^{2}-2R_{R}r_{i}\cos\left(\frac{\pi }{2}-\frac{\beta }{2}+\theta_{i}\right)}$$

in which $\theta_{i}=\theta_{0}+\Delta \theta_{i}$, in far field condition. We have $R_{T}>r_{i}$, $R_{R}>r_{i}$, then (A1) can be approximately expressed by

(A2) $$R\approx R_{T}-r_{i}\cos\left(\frac{\pi }{2}+\frac{\beta }{2}+\theta_{i}\right)+R_{R}-r_{i}\cos\left(\frac{\pi }{2}-\frac{\beta }{2}+\theta_{i}\right)$$

in which $x_{i}=r_{i}\cos\theta_{0},y_{i}=r_{i}\sin\theta_{0}$, then (A2) can be rewritten as

(A3) $$R\approx R_{T}+R_{R}+2\cos\frac{\beta }{2}\left[y_{i}\cos\Delta \theta_{i}+x_{i}\sin\Delta \theta_{i}\right]$$

### Appendix A.2. Derivation of the Complex Amplitude in (19) and (20)

Take (2) into (9) and (10), we will get the amplitude shown as follows:

(A4) $${\tilde{\sigma }}_{1}(x_{i},y_{i})=\sigma_{i}\mathrm{rect}\left(\frac{\hat{t}-\frac{R}{c}}{T_{p}}\right)\cdot \exp\left(\frac{j2\pi \gamma \left(\hat{t}-T_{ref}\right)}{c}x_{i}wt_{m}\right)$$

(A5) $${\tilde{\sigma }}_{2}(x_{i},y_{i})=\sigma_{i}\mathrm{rect}\left(\frac{\hat{t}-\frac{R}{c}}{T_{p}}\right)\cdot \exp\left(\frac{j2\pi \gamma \left(T_{ref}-\hat{t}\right)}{c}xliwt_{m}\right)$$
